# 500例肺癌患者首诊症状及其影响因素分析

**DOI:** 10.3779/j.issn.1009-3419.2018.05.09

**Published:** 2018-05-20

**Authors:** 昕 张, 镨元 邢, 学志 郝, 峻岭 李

**Affiliations:** 100021 北京，国家癌症中心/中国医学科学院北京协和医学院肿瘤医院 National Cancer Center/Cancer Hospital, Chinese Academy of Medical Sciences and Peking Union Medical College, Beijing 100021, China

**Keywords:** 肺肿瘤, 症状, 诊断, 治疗, 影响因素, Lung neoplasms, Symptom, Diagnosis, Treatment, Influencing factors

## Abstract

**背景与目的:**

肺癌的发病率与死亡率不断升高，本研究探讨初诊肺癌患者临床特征，特别是首诊症状对诊断及后续治疗选择的影响。

**方法:**

回顾性分析自2017年3月-2017年5月我院胸内科门诊就诊的500例肺癌患者，分析其临床特征包括首发症状、分期、生物标志物、病理等对后续治疗选择影响。

**结果:**

女性（53.3%）、腺癌（74.4%）、吸烟者（58%）多见，大部分患者（98.2%）体力状态评分为0分-1分。生物标志物检测58.2%（*n*=291），其中表皮生长因子受体（epidermal growth factor receptor, *EGFR*）突变61.2%（178/291），间变性淋巴瘤激酶（anaplasticlymphoma kinase, *ALK*）融合基因阳性4.1%（12/291）。不同吸烟状况、是否有症状、病理分型、疾病分期和是否有突变是影响后续治疗的主要因素。

**结论:**

就诊时有典型症状的患者确诊时间更短，吸烟状况、肺癌相关症状、病理、疾病分期及突变状况是影响后续治疗的主要因素。

## Abstract (en)

**Background and objective:**

As the morbidity and mortality in lung cancer keep raising, we are here to discuss the effect of clinical features especially the initial symptomon on diagnosis and follow-up treatment of newly diagnosed lung cancer patients.

**Methods:**

The clinical features of the 500 patients with lung cancer in our hospital from March, 2017 to May, 2017 were analyzed retrospectively, including the initial symptom, stage, biomarkers, pathology, etc.

**Results:**

There were 266 famle (53.3%), 372 adenocarcinoma (74.4%), 285 smokers (58%), status score of most patients (98.2%) was 0-1. 58.2% (*n*=291) of all the patients got biomarkers test, of which epidermal growth factor receptor (*EGFR*) mutations was 61.2%(178/291), anaplasticlymphoma kinase (*ALK*) fusion gene positive was 4.1% (12/291). Smoking status, initial symptom, pathological typing, TNM staging and *EGFR* mutation were the main factors affecting follow-up treatment.

**Conclusion:**

Patients with typical symptoms have shorter diagnosis time. Smoking status, lung cancer-related symptoms, pathology, TNM staging and *EGFR* mutation status are the main factors that affect the follow-up treatment.

全世界范围内，肺癌都是影响人类健康的重大问题，其5年生存率仅为约15%^[1, 2]^。近年来，中国的肺癌发病率和死亡率急剧上升，预估每年诊断的新发病例733, 300例，死亡610, 200例^[3]^。大多数肺癌患者在诊断时疾病已达晚期，失去了治愈的机会。本研究回顾性分析2017年3月-2017年5月于中国医学科学院肿瘤医院确诊为肺癌的患者，统计分析其临床特征、诊断和治疗方面的差异及影响因素，现报告如下。

## 1 资料与方法

### 1.1 入组标准

① 经组织病理学或细胞学诊断明确的肺癌患者；②临床资料完整，包括患者人口学特征，如年龄、性别、病理诊断、体力状态评分（performance status, PS）、肿瘤-淋巴结-转移（tumor-node-metastasis, TNM）疾病分期和吸烟状况；③影像学检查全面，包括但不限于胸部电子计算机断层（computed tomography, CT）扫描（平扫或增强）、针对骨转移的全身放射性核素骨显像、颅脑磁共振成像（magnetic resonance imagin, MRI）检查、全身正电子发射计算机断层显像（positron emission tomography-computed tomography, PET-CT）检查等；④在本中心接受首程治疗；⑤本研究不纳入复查病例。

### 1.2 数据分析

本研究收集了2017年3月-2017年5月就诊于中国医学科学院肿瘤医院门诊，且确诊为肺癌的患者的病历资料。收集的信息资料包括患者人口学特征，如年龄、性别、病理诊断、体力状态评分PS、TNM疾病分期和吸烟状况。肿瘤诊断时间定义为首发症状日期到病理确诊的时间。该分析中还包括了肿瘤的驱动基因，主要为目前已用于临床的表皮生长因子受体（epidermal growth factor receptor, *EGFR*）基因突变，间变性淋巴瘤激酶（anaplastic lymphoma kinase, *ALK*）重排，原癌基因1（c-ros oncogene 1, *ROS1*）重排和鼠类肉瘤病毒癌基因（Kirsten rat sarcoma viral oncogene homolog, *Kras*）突变。原癌基因（rearranged during transfection, *RET*）重排等目前未用于临床诊疗常规的少见肿瘤驱动基因不在本研究分析之列。

### 1.3 统计方法

统计软件为SPSS 20.0版（SPSS, Chicago, IL, USA）。本研究采用描述性统计方法进行分析。人口特征通过连续变量的方式和标准偏差以及分类变量的频率和百分比来总结。两样本的检验采用*t*检验或非参数检验，余用卡方检验。双侧*P* < 0.05为具有统计学意义。

## 2 结果

### 2.1 一般临床特征

2017年3月-2017年5月期间，共收集到500例明确诊断为肺癌的新发肿瘤患者的信息。所有患者的人口学和疾病特征见表 1。

**1:** 患者特征 Basic characteristics of the patients

Characteristics	Items	*n* (%)
Gender	Male	233 (46.7)
	Female	266 (53.3)
Age (yr)	Median (range)	59 (28-84)
	< 55	163 (32.6)
	55-70	271 (54.2)
	> 70	66 (13.2)
Smoking history	No	206 (42.0)
	Yes	285 (58.0)
Symptom	No	176 (35.2)
	Yes	324 (64.8)
Histology type	Adenocarcinoma	372 (74.4)
	Squamous cell carcinoma	85 (17.0)
	Small cell lung cancer	31 (6.2)
	Adenosquamous carcinoma	4 (0.8)
	Other	7 (1.4)
Stage	Ⅰ	167 (33.4)
	Ⅱ	52 (10.4)
	Ⅲa	76 (15.2)
	Ⅲb	29 (5.8)
	Ⅳ	176 (35.2)
Performance status	0	414 (82.8)
	1	77 (15.4)
	2	9 (1.8)
Biomarkers test	Yes	291 (58.2)
	No	209 (41.8)

所有患者年龄在28岁-84岁之间，中位年龄58岁。大多数患者集中在55岁-70岁这一年龄段（*n*=271, 54.2%）。性别比例方面，女性患者高于男性患者（53.3% *vs* 46.7%）。大部分患者有吸烟史，占整体人群的58%。病理分型分别为腺癌（*n*=372, 74.4%）、鳞癌（*n*=85, 17.0%）、小细胞肺癌（*n*=31, 6.2%）、腺鳞癌（*n*=4, 0.8%）和其他（*n*=7, 1.4%）。疾病分期分别为Ⅰ期（*n*=167, 33.4%）、Ⅱ期（*n*=52, 10.4%）、Ⅲa期（*n*=76, 15.2%）、Ⅲb期（*n*=29, 5.8%）和Ⅳ期（*n*=176, 35.2%）。大部分患者就诊时体力状态良好（PS 0-1, 98.2%）。

58.2%的患者（*n*=291）接受了生物标志物分析检测，其中101例（34.7%）患者所有的生物标志物检测均为阴性。*EGFR*突变是本研究人群中最常见的（61.2%, 178/291）驱动基因，包括19位点突变（33.3%）、21位点突变（30.2%）、18位点突变（1.0%）、20位点突变（1.7%）和双突变（2.1%）。291例患者中12例（4.1%）为*ALK*融合基因阳性。

### 2.2 症状、检测方法与诊断

324例（64.8%）患者就诊时出现临床症状，包括干咳（*n*=174, 34.8%）、痰血（*n*=34, 6.8%）、胸痛（*n*=36, 7.2%）、喘憋（*n*=30, 6.0%）、胸部以外肢体疼痛（*n*=25, 5.0%）、中枢神经系统症状（*n*=7, 1.4%）、颈部包块（*n*=12, 2.4%）和声嘶（*n*=6, 1.2%）。176例（*n*=176, 35.2%）患者通过查体发现肿瘤。

疾病的中位诊断时间为40 d。性别、年龄、是否吸烟、是否有症状、病理分型、疾病分期等临床特征的差异对确诊时间无明显影响（表 2）。进一步分析临床症状对诊断时间的影响，结果显示，不同首发症状间确诊时间有统计学差异，提示不同首发症状间确诊时间不完全相同（表 3）。进一步两两比较结果显示，首发症状为痰血、以及查体发现的患者确诊时间小于首发症状为干咳、胸痛、喘憋、胸部以外肢体疼痛和颈部包块的患者确诊时间。

**2:** 确诊时间[中位数（95%CI）]及影响因素分析 diagnosis time [median (95%CI)] and analysis of influencing factors

	Characteristics	*n*	Diagnosis time (d)	*t*/*χ*^2^	*P*
Gender	Male	266	42.5 (25-90)	0.149	0.882
	Female	233	36 (27-90)		
Age (yr)	< 70	434	40 (24-90)	1.014	0.311
	≥70	66	49.5 (30-116.3)		
Smoking	Yes	205	45 (24.5-90)	0.841	0.401
	No	286	39 (28-90)		
Symptom	Yes	324	60 (30-100)	0.293	0.770
	No	176	30 (20-67.5)		
Histology type	Adenocarcinoma	372	41 (26.5-94.5)	5.367	0.068
	Squamous cell carcinoma	85	45 (29-90)		
	SCLC	31	30 (21-60)		
Stage	Ⅰ-Ⅲa	295	40 (24-90)	0.191	0.849
	Ⅲb-Ⅳ	205	50 (28.5-90)		
Driver gene	Positive	196	42 (24-90)	0.379	0.705
	Negative	101	45 (30-120)		
SCLC: small cell lung cancer.					

**3:** 不同首发症状与肺癌确诊时间 Initial symptoms and diagnosis time

Characteristics	*n*	Diagnosis time [median (P25-P75)]	*χ*^2^	*P*
Cough	174	60 (30-120)	41.519	< 0.001
Hemoptysis	34	26.5 (15-38.25)		
Chest pain	36	50 (29.25-120)		
Shortness of breath	30	47 (22.5-90)		
Body pain (except chest)	25	60 (30-90)		
Symptom of central nervous system	7	90 (18-180)		
Neck lump	12	70 (27-90)		
Physical examination	176	30 (20-67.5)		
Hoarseness	6	60 (24.25-112.5)		

### 2.3 治疗

采用卡方检验比较患者临床特征间治疗方法分布差异，结果表 4所示：治疗方法根据不同吸烟状况、是否有症状、病理分型、疾病分期和是否有突变存在统计差异。不吸烟者更多采用靶向治疗；有症状者更多采用化疗和靶向，无症状者多采用手术治疗；小细胞肺癌多采用化疗；疾病分期为Ⅲb期-Ⅳ期患者更多采用化疗和靶向治疗，疾病分期为Ⅰ期-Ⅲa期者多采用手术治疗；有突变者多采用靶向治疗，无突变者多采用化疗。

**4:** 确诊后常见治疗方法及影响治疗方法的因素分析 Treatment after diagnosis and analysis of influencing factors [*n* (%)]

Factors	Characteristic	Surgery	Chemotherapy	Radiotherapy	Chem+radio	Targeted therapy	*χ*^2^	*P*
Gender	Male	151 (57.2)	67 (25.4)	7 (2.7)	7 (2.7)	32 (12.1)	8.534	0.074
	Female	125 (53.6)	48 (25.4)	6 (2.6)	4 (1.7)	50 (21.5)		
Age (yr)	< 70	235 (54.4)	104 (24.1)	11 (2.5)	8 (1.9)	74 (17.1)	4.328	0.363
	≥70	41 (62.1)	11 (16.7)	2 (3.0)	3 (4.5)	9 (13.6)		
Smoking	Yes	121 (59.6)	54 (26.6)	7 (3.4)	6 (3.0)	15 (7.4)	19.380	0.001
	No	153 (53.5)	60 (21.0)	6 (2.1)	5 (1.7)	62 (21.7)		
Symptom	Yes	137 (42.4)	100 (31.0)	10 (3.1)	7 (2.2)	69 (21.4)	65.692	< 0.001
	No	139 (79.4)	15 (8.6)	3 (1.7)	4 (2.3)	14 (8.0)		
Histology type	Aden	209 (56.3)	70 (18.9)	9 (2.4)	4 (1.1)	79 (21.3)	72.875	< 0.001
	SCC	59 (67.1)	19 (22.4)	4 (4.7)	3 (3.5)	2 (2.4)		
	SCLC	5 (16.7)	22 (73.3)	0 (0.0)	2 (6.7)	1 (3.3)		
Stage	Ⅰ-Ⅲa	267 (90.5)	17 (5.8)	4 (1.4)	1 (0.3)	6 (2.0)	363.663	< 0.001
	Ⅲb-Ⅳ	9 (4.4)	98 (48.3)	9 (4.4)	10 (4.9)	77 (37.9)		
Driver gene	Positive	104 (53.1)	25 (12.8)	2 (1.0)	1 (0.5)	64 (32.7)	38.876	< 0.001
	Negative	61 (61.0)	23 (23.0)	5 (5.0)	5 (5.0)	6 (6.0)		
Aden: adenocarcinoma; SCC: squamous cell carcinoma.								

## 3 讨论

随着人口老龄化以及大气污染的加重，我国肺癌的发病率和死亡率逐年增高，为患者家庭和社会带来了巨大的经济负担。随着诊断技术的进步、筛查的普及以及对肿瘤发生机制认识的加深，近年来肺癌的诊治发生了巨大的变化。

本研究结果显示，女性肺癌患者的所占比例相对较高（53.3%）。肺癌在男性中一直较女性更为常见^[1-3]^。然而，这种性别差异持续下降。吸烟和被动吸烟是女性患者比例升高的主要原因，此外，在密闭空间中烹饪也被认为对中国女性肺癌发病率的增加有影响^[4, 5]^。

腺癌是本研究中最常见的组织学亚型，占所有患者的74.4%。在相当一段时间内，组织学亚型（包括小细胞肺癌、鳞癌和非鳞肺癌）已足以指导肺癌患者的治疗。但近年来，随着肿瘤分子生物学研究的深入，出现了针对驱动基因的靶向治疗方案，使得生物标志物检测愈加重要。本研究中，291例（58.2%）患者进行了生物标志物检测，提示在真实世界的临床实践中，针对驱动基因的分子生物学检测已成为肺癌的诊疗常规。本研究中仅观察到*EGFR*突变（*n*=178）和*ALK*融合（*n*=12）两种驱动基因。*EGFR*突变发生率（61.2%）与既往报告的亚洲患者*EGFR*突变率基本一致。PIONEER研究分析了亚洲1482例患者的肿瘤，*EGFR*突变发生率为22%-62%^[6]^。与既往研究报道相符，21 L858R和19 Del是本研究中*EGFR*突变的主要亚型^[6-8]^，18、20位点突变和双突变仅占4.8%。*ALK*融合在非小细胞肺癌中相对罕见，在未选择群体中其突变率为1.5%-6.7%，而在非吸烟患者中，ALK阳性率可高达22%^[9-14]^。本研究中，4.1%的患者出现*ALK*重排，阳性率与既往研究一致。

本研究500例患者中，Ⅰ期、Ⅱ期、Ⅲa期、Ⅲb期和Ⅳ期患者分别占33.4%、10.4%、15.2%、5.8%和35.2%，早期患者相对较多（Ⅰ期-Ⅲa期，59%），这与大多数肺癌患者在诊断时患有晚期疾病的一般认知相反。这可能是因为中早期疾病患者经过初诊医师的推荐转诊，以便接受根治性治疗，晚期患者多在各地市及县级医院首诊，在当地治疗。此外，相当一部分患者（35.2%）通过查体发现肺癌，这可能也是本研究中早期患者占比例较高的原因之一。

性别、年龄、吸烟、症状、病理分型、疾病分期等临床特征的差异对确诊时间无明显影响。64.8%的患者因出现临床症状就诊进而发现肺癌，临床症状主要以干咳、痰血、胸痛和喘憋等呼吸系统相关症状为主。其中首发症状为痰血的患者确诊时间明显小于出现其他首发症状的患者的确诊时间。本研究中35.2%的患者通过查体发现肺癌，提示筛查在肺癌诊断中发挥重要作用。大规模、随机、前瞻性国家肺癌筛查试验（National Lung Screening Trial, NLST）证实，高危人群（根据年龄和吸烟史）每年接受低剂量CT（low-dose CT, LDCT）扫描，可降低肺癌死亡率达20.0%，表明高风险人群的LDCT筛查有助于发现早期疾病从而降低肺癌死亡率^[15]^。然而，这种长期筛查需要考虑成本效益问题，McMahon等^[16]^报道，LDCT筛查可将10年肺癌死亡率降低18%-25%，每个质量调整生命年的成本为126, 000美元-269, 000美元。

随着新的治疗药物不断应用于临床，晚期肺癌患者生存期和生活质量都得到了提高。然而早期诊断接受根治性治疗仍然是肺癌患者得到痊愈的最佳手段。本研究提示，咳血是最易于引起患者警觉并积极就诊的症状，而咳嗽、喘憋、胸痛以及其他症状容易被患者忽视。健康查体是肺癌患者得到早期诊断治疗的大好机会，本研究提示仍有部分病例在查体发现肺部病灶后没有进一步检查和积极随诊，直到出现症状后才再次就诊，错过了最佳治疗时机。这一方面说明提高民众的就医意识、进行防癌抗癌科普宣教，仍旧任重道远，另一方面也说明首次接诊医生的重要性。随着生活水平的提高，患者对于自己所接受的医疗服务有着越来越高的期望，而肺癌发病率死亡率还在不断增高，这一切都对首诊医生提出更高的要求。

本研究有若干局限性：该研究样本量较小且为回顾性研究；单中心研究可能导致数据收集的偏倚；本研究未收集生存数据，因此无法评价诊断和治疗对患者疾病结局的影响。但总体来说，本研究通过观察500例初治患者疾病特征、诊断和治疗方面的差异，反映了精准医疗时代多学科综合诊治下的临床实践现状：女性患者比例升高，但还需要更大样本量的流行病学研究证实；相对于干咳、喘憋、胸痛等非特异性症状，一些相对较为严重的特异性症状，如痰血，更容易引起人们的重视，从而进行全面检查进而发现肺癌；早期筛查和基于肿瘤分子生物学的驱动基因检测正趋于常规化并在肺癌诊断治疗中发挥重要作用。
